# Multiple associated anomalies in a single patient of duodenal atresia: a case report

**DOI:** 10.1186/1757-1626-1-215

**Published:** 2008-10-06

**Authors:** Bilal Mirza, Lubna Ijaz, Muhammed Saleem, Afzal Sheikh

**Affiliations:** 1 Department of paediatric surgery, children hospital, Institute of child health, ferozepur road, Lahore, Pakistan

## Abstract

**Background:**

Duodenal atresia is a common cause of intestinal obstruction in neonates. It is associated with other congenital anomalies like Down's syndrome, annular pancreas etc.

**Case presentation:**

We present a case of a two days old male baby presented to us with bilious vomiting since birth. It was associated with Down's syndrome, Annular pancreas and Malrotation.

**Conclusion:**

Duodenal atresia is associated with other congenital anomalies but more than one congenital anomalies in a single patient is very rare.

## Background

The duodenum is the most common site of intestinal obstruction accounting for nearly half of all cases [1]. The incidence of duodenal atresia is estimated as 1 in 6000–10000 births [2]. Embryological basis for etiology of duodenal atresia are thought to be due to errors of recanalization [3]. Approximately half of all infants with duodenal atresia or stenosis will also have a congenital anomaly of another organ system [4].

Duodenal atresia is associated with Down's syndrome (30%), annular pancreas (23%), congenital heart disease (22%), malrotation (20%), oesophageal atresia (8%), others (20%) [5].

The mainstay of treatment is surgical intervention. Duodenoduodenostomy is most frequently performed operation.

We report a case of duodenal atresia associated with more than one congenital anomalies.

## Case presentation

A male baby 2 days old presented to us for bilious vomiting since birth. On examination patient has features of Down's syndrome and no other obvious anomaly. A nasogastric tube is passed and 40–50 ml bilious fluid aspirated. Xrays abdomen erect film showed a double bubble sign. Provisional diagnosis of duodenal atresia made and patient was optimized for surgery 1, 2.

**Figure 1:**
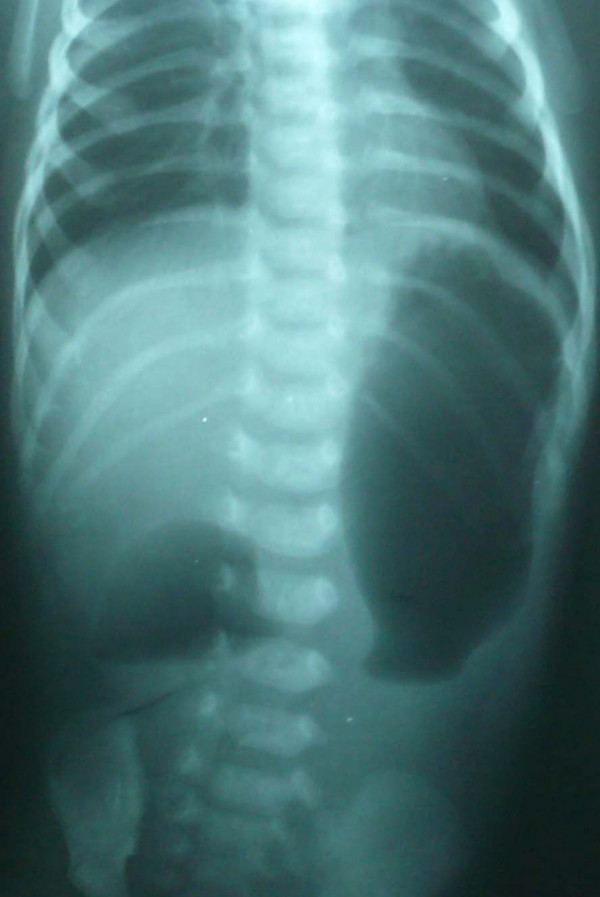
X-rays radiograph abdomen (erect posture) showing a double bubble sign indicative of duodenal obstruction.

**Figure 2:**
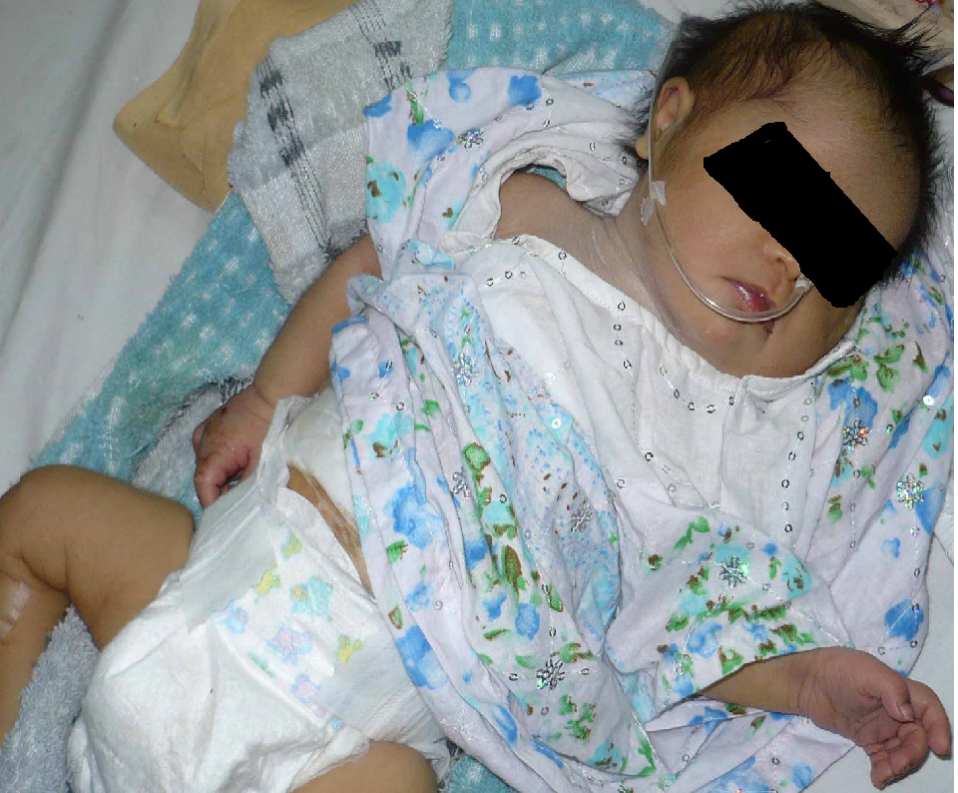
Picture of the patient with down syndrome manifestations.

At operation we found duodenal atresia, annular pancreas and malrotaion of gut.

Duodenoduodenostomy was peformed. Recovery of patient was uneventful.

## Conclusion

Duodenal atresia is frequently associated with other congenital anomalies but combination of anomalies in a single patient is very rare. This report illustrates a rare setting in which three associated congenital anomalies were present in a single patient of duodenal atresia.

## Consent

Written informed consent was obtained from the parents for publication of this case report.

## Competing interests

The authors declare that they have no competing interests.

## Authors' contributions

BM presented the case history, researched the topic and helped draft the manuscript. LI reviewed the literature and drafted the manuscript. MS was the supervising consultant paediatric surgeon who supervised the surgery. AS is head of department helped and guided for preparation of case report. All authors read and approved the final manuscript.
